# Expanding the known phenotype of Mullegama–Klein–Martinez syndrome in male patients

**DOI:** 10.1038/s41439-021-00169-3

**Published:** 2021-09-27

**Authors:** Fiona Freyberger, Tomislav Kokotović, Goran Krnjak, Sanda Huljev Frković, Vanja Nagy

**Affiliations:** 1 Ludwig Boltzmann Institute for Rare and Undiagnosed Disorders, Vienna, Austria; 2 Department of Neurology, Medical University of Vienna, Vienna, Austria; 3 CeMM - Research Center for Molecular Medicine of the Austrian Academy of Sciences, Vienna, Austria; 4 Department of Pediatrics, Varazdin General Hospital, Varazdin, Croatia; 5 Department of Paediatrics, Division for Genetics and Metabolism, University of Zagreb School of Medicine, University Hospital Centre Zagreb, Zagreb, Croatia

**Keywords:** Neurodevelopmental disorders, Neurological disorders

## Abstract

Here, we report a novel case of a male patient with a hemizygous missense variant in *STAG2* (p.Tyr159His) resulting in Mullegama–Klein–Martinez syndrome (MKMS), a rare X-linked cohesinopathy. He shares distinct clinical features with a previously reported male patient carrying the STAG2 variant p.Tyr159Cys, suggesting that this phenotype is determined by the position of the mutation. Additionally, our patient exhibits symptoms not previously associated with MKMS, expanding the known clinical phenotype of this rare disease.

Mullegama–Klein–Martinez syndrome (MKMS), OMIM#301022, is a rare disease caused by deleterious variants in the *STAG2* gene, which codes for the cohesin subunit, Stromal Antigen 2 (STAG2). MKMS is associated with diverse clinical symptoms, including developmental delay, intellectual disability, craniofacial abnormalities, and brain malformations. STAG2 is a component of the multimeric cohesion complex that regulates sister chromatid cohesion during mitosis and meiosis; it also regulates DNA replication, DNA repair, and transcription^1^. The protein is predicted to consist of a STAG domain^2^ and a stromalin conservative domain (SCD) (https://www.uniprot.org/uniprot/Q8N3U4). Mutations within *STAG2* have been identified in several different cancers and are highly constrained toward *loss of function* with a pLI = 1 and o/e = 0.02 (https://gnomad.broadinstitute.org/gene/ENSG00000101972?dataset=gnomad_r2_1). MKMS is inherited in an X-linked manner and has highly variable phenotypes. Among the 18 patients reported in total, 15 were female, and 3 were male. In females, 13 of the 15 variants were truncating, 1 variant led to a missense change, and 1 variant was in a splicing area, while in males, only missense variants were reported (Yuan: de novo; Mullegama: de novo; Soardi: maternal)^1–8^. As the disorder is highly variable in phenotype and the clinical information on male patients has been limited to only three cases, there is a need for additional, more detailed case reports to shed light on all aspects of the disorder, including possible sex-related differences. Here we report the 19th case of MKMS, a 10-year-old male patient with a hemizygous missense *STAG2* variant (GRCh37/hg19: chrX:123179026, c.475T>C, p.Tyr159His; NC_000023.11:g.124045176T>C) with previously unreported clinical features that expand the known manifestation of the disorder.

The patient was resuscitated at birth and showed hypotonia and decreased response to external stimuli (Table 1). Postnatally, he was referred to a pediatric geneticist because of his dysmorphic features. He was described to have dolichocephaly, coarse facial features, a narrow bifrontal diameter, a high forehead, a prominent metopic suture, a broad nasal bridge, a bulbous nose, antimongoloid palpebral fissures, thick lips, and a high-arched palate (Fig. 1A). His hands and feet as well as his fingers and toes were broad, with soft dorsal surfaces; the nails were deeply inserted; and the joints of the hands and feet were hyperextensible. The patient’s metabolic findings and karyotype were normal.

**Table 1:** Clinical features of the MKMS patients.

Clinical features		Patient	P1	P2	P3	P4	P5	P6	P7	P8	P9	P10	P11	P12	P13	P14	P15	P16	P17	P18	P19
		Sex	F	F	F	F	F	F	F	F	F	F	F	F	F	F	F	M	M	M	M
		cDNA change AA change	c.418C>T p.Q140*	c.1605T>A p.C535*	c.1811G>A p.R604Q	c.1658_1660 delinsT p.K553Ifs*6	c.205C>T p.R69*	c.3097C>T p.R1033*	c.2229G>A p.W743*	c.1840C>T p.R614*	c.205C>T p.R69*	c.436C>T p.R146*	c.775C>T p.R259*	c.3034C>T p.R1012*	c.2898_2899del p.E968Sfs*15	c.2533+1G>A	c.1639delG p.V547Cfs*29	c.3027A>T p.K1009N	c.980G>A p.S327N	c.476A>G p.Y159C	c.475T>C p.Y159H
Growth abnormalities		IUGR	−	+	−	−	−	NR	NR	NR	+	NR	NR	NR	NR	NR	NR	NR	NR	−	+
		Failure to thrive	NR	+	+	−	NR	NR	NR	NR	NR	NR	NR	NR	NR	NR	NR	+	+	+	+
		Short stature	NR	+	+	+	+	NR	NR	NR	NR	NR	NR	NR	NR	NR	NR	NR	+	+	+
Dysmorphic features and malformations	Neurocranium	Pathological brain MRI	NR	NR	NR	+	+	+	+	+	+	+	+	+	+	+	+	−	NR	+	+
		Microcephaly	−	+	+	+	+	NR	+	+	+	+	−	+	+	+	NR	+	NR	−	−
		Brachicephaly	−	+	NR	−	NR	NR	NR	NR	NR	NR	NR	NR	NR	NR	NR	NR	NR	NR	−
	Eyes	Long eyelashes	NR	+	+	−	NR	NR	NR	NR	NR	NR	NR	NR	NR	NR	NR	NR	NR	−	−
		Strabismus	NR	+	NR	NR	NR	NR	NR	NR	NR	NR	NR	NR	NR	NR	NR	NR	NR	−	−
	Nose	Anteverted nares	NR	NR	+	+	NR	NR	NR	NR	NR	NR	NR	NR	NR	NR	NR	NR	NR	−	−
		Broad bridge	−	−	+	+	NR	NR	NR	NR	NR	NR	NR	NR	NR	NR	NR	NR	NR	+	+
		Bulbous tip	−	NR	+	+	NR	NR	NR	NR	NR	NR	NR	NR	NR	NR	NR	NR	NR	+	+
	Ears	Low set	NR	NR	−	+	+	NR	NR	NR	−	+	−	+	NR	NR	NR	NR	NR	+	+
		Dysmorphic	+	+	+	+	+	NR	NR	NR	−	NR	−	NR	NR	NR	NR	NR	+	+	+
	Lips and mouth	Long philltrum	NR	+	+	−	NR	NR	+	NR	NR	NR	NR	NR	NR	NR	NR	NR	NR	−	−
		Thin upper lip	+	+	+	−	NR	NR	NR	NR	NR	NR	NR	NR	NR	NR	NR	NR	NR	+	−
		Downturned mouth	NR	NR	+	−	NR	NR	NR	NR	NR	NR	NR	NR	NR	NR	NR	NR	NR	−	−
		Cleft palate	−	−	NR	−	+	+	+	NR	+	NR	NR	+	NR	NR	NR	−	+	+	−
		High arched palate	−	−	NR	+	NR	NR	NR	NR	NR	NR	NR	NR	NR	NR	NR	NR	NR	NR	+
		Micrognathia	NR	+	+	+	+	NR	NR	NR	+	NR	NR	NR	NR	NR	NR	NR	NR	−	+
	Skin and adnexa	Marmorization	NR	+	NR	−	NR	NR	NR	NR	NR	NR	NR	NR	NR	NR	NR	NR	NR	−	−
		Low hairline	NR	+	NR	NR	+	NR	NR	NR	NR	NR	NR	NR	NR	NR	NR	−	NR	NR	+
		Hirsutism	NR	+	NR	−	NR	NR	NR	NR	NR	NR	NR	NR	NR	NR	NR	NR	NR	−	−
		Hipoplastic nails	+	NR	NR	+	NR	NR	NR	NR	NR	NR	NR	NR	NR	NR	NR	NR	NR	−	−
	Hands and feet	Fifth finger clindactily	NR	+	NR	−	+	NR	NR	NR	NR	NR	NR	NR	NR	NR	NR	+	−	NR	−
		Single palmar crease	−	+	NR	−	NR	NR	NR	NR	NR	NR	NR	NR	NR	NR	NR	NR	NR	NR	−
		Second–third toe syndactily	−	+	NR	−	+	NR	NR	NR	NR	NR	NR	NR	NR	NR	NR	−	NR	−	−
	Other skeletal	Scoliosis	+	NR	NR	+		NR	NR	+	NR	NR	NR	NR	NR	NR	NR	−	−	+	+*
		Rib fusion	+	NR	+	+	NR	NR	NR	NR	NR	NR	NR	NR	NR	NR	NR	NR	NR	−	−
		Vertebral abnormalities	+	NR	+	+	NR	NR	+	NR	−	+	NR	+	NR	NR	NR	NR	NR	−	−
Other systems		Renal tract abnormalities	−	NR	NR	−	NR	NR	NR	NR	NR	NR	NR	NR	NR	NR	NR	NR	NR	+	−
		Pulmonary hypoplasia	NR	NR	+	−	NR	NR	NR	NR	NR	NR	NR	NR	NR	NR	NR	NR	NR	−	−
		Congenital diaphragmatic hernia	+	NR	+	−	NR	NR	NR	NR	NR	NR	NR	NR	NR	NR	NR	NR	NR	NR	−
		GERD	+	+	+	−	NR	NR	NR	NR	NR	NR	NR	NR	NR	NR	NR	NR	NR	−	−
		Congenital heart failure	+	NR	−	NR	+	+	−	NR	+	+	+	NR	−	+	NR	−	−	+	−*
		Abnormal echocardiogram	+	NR	NR	NR	+	NR	NR	NR	NR	NR	NR	NR	NR	NR	NR	NR	NR	−	−
Neuro functional		NDD	+	+	+	+	+	NR	+	+	+	NR	+	NR	+	NR	+	+	+	+	+
		ID	NR	+	+	+	+	NR	+	NR	NR	NR	+	NR	NR	NR	NR	NR	+	+	+
		Seizures	+	NR	NR	+	NR	NR	NR	NR	NR	NR	NR	NR	NR	NR	+	NR	NR	−	+
		Hypotonia	NR	+	+	+	NR	NR	NR	NR	NR	NR	NR	NR	NR	NR	NR	+	NR	+	+
		Hearing loss	NR	+	NR	−	+	NR	+	NR	−	−	−	NR	NR	NR	+	−	+	−	−
		Behavioral problems	NR	+	NR	+	−	NR	+	NR	NR	NR	NR	NR	NR	NR	NR	+	NR	−	−

Additional clinical features: P1—myoclonisms, hypoplastic left heart, VSD, CA, vertebral clefts; P2—low, posterior hairline; P4—irritability in behavior; P5—ADHD, dysgenesis of the splenium of the corpus callosum, interventricular subarachnoid cyst, subgaleal hematoma, left facial palsy, mild left pelviectasis; P6—pregnancy was terminated at 21 gestational weeks because of multiple fetal abnormalities; P7—white matter hypoplasia, speech delay, amblyopia; P8—cystic pituitary lesion, sacral dimple, CDH; P9—left heart hypoplasia, VSD, CA; P10—cyclopia, absent nose, duodenal atresia; P11—septo-optic dysplasia, left hip dysplasia, bilateral optic nerve hypoplasia, P12—GERD, P14—left heart hypoplasia, DORV; P15—focal seizures with secondary generalization, gelastic seizures (age of onset 6 years; drug resistant), dysmorphic features; P16—high hairline, foot polydactyly; P17—large nose, prominent ears, frontal baldness; P18—minimal PFO, normal on follow-up, single kidney, ectopic posterior pituitary, short pituitary stalk; P19—dolichocephaly, broad hands and feet with soft dorsum and deeply inserted nails, hyperextensible joints, polymicrogyria, hypotrophic presplenial part of corpus callosum, ectopic posterior pituitary, thin pituitary stalk.

P1–P3: Yuan et al.^4^; P4–P5: Mullegama et al.^1^; P6–P8: Aoi et al.^2^; P9–P11: Lan Yu et al.^9^; P12–P14: Kruszka et al.^6^; P15: Epilepsy Genetics Initiative^8^; P16: Mullegama et al.^5^; P17: Soardi et al.^3^; P18: Yuan et al.^4^; P19: this study.

**Fig. 1:**
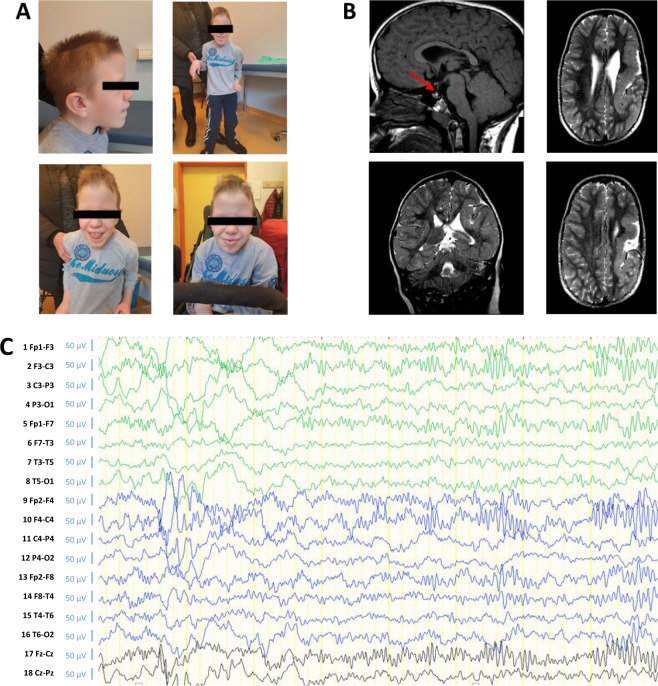
Patient phenotype and EEG. **A** Clinical photos of the patient at 11 years of age. **B** MRI images taken at 7 years of age. The upper left panel shows presplenial corpus callosum hypoplasia and an ectopic neurohypophysis positioned along the caudal part of the infundibulum (indicated by arrow, T1 sequence). The other three images show an extensive frontotemporoparietal area of cortical thickening consistent with polymicrogyria; the left parietal lobe had a deep sulcus impressed into the lateral ventricle (T2 sequence). **C** Bilateral longitudinal montage, sensitivity 7 µV/mm. Asleep, N2 stage. Continuous beta activity was most pronounced over frontocentral regions. The EEG was taken at 7 years of age.

Initial magnetic resonance imaging (MRI), performed at the age of 10 months, revealed left-sided perisylvian polymicrogyria associated with a hypoplastic corpus callosum. At the age of 7 years, lamotrigine therapy was started due to an episode of generalized tonic–clonic seizures. The patient’s electroencephalogram showed continuous beta activity bilaterally over the frontocentral regions, but there was no epileptiform activity (Fig. 1C).

Due to epilepsy, a second brain MRI was performed at the age of 7 years, showing an extensive frontotemporoparietal area of cortical thickening consistent with polymicrogyria (Fig. 1B); the left parietal lobe had a deep sulcus impressed into the lateral ventricle. Additionally, a small area of T2/FLAIR hyperintensity was described in the right peritrigonal region as a consequence of perinatal hypoxia. The lateral ventricles were moderately dilated, without signs of hypertensive hydrocephalus. The presplenial part of the corpus callosum was hypotrophic. The pineal gland was medially positioned with a thin infundibulum and a small, medially positioned adenohypophysis. An ectopic neurohypophysis was positioned along the caudal part of the infundibulum. Ophthalmological findings showed an atrophic retinal and uveal scar of the right eye. Additionally, echocardiography revealed a persistent foramen ovale with a small, hemodynamically insignificant shunt; however, by the next follow-up 1 year later, it was closed. Currently, at 10 years of age, the patient has short stature. He has motoric problems such as coarse and uncoordinated fine motor skills, truncal hypotonicity with fluctuation to hypertonus, and increased tendon reflexes. He cannot walk, needs assistance standing, and has severe left-sided planovalgus and right-sided equinovarus (Table 1). His speech development is delayed; he is able to communicate but has difficulties in pronunciation.

For the genetic analysis of the patient and family, genomic DNA (gDNA) was isolated from blood samples using the Qiagen’s DNeasy Blood and Tissue Kit. The patient’s gDNA was submitted for whole-exome sequencing (Illumina HiSeq3000) with an average target coverage of 91×. Bioinformatical analysis of the raw sequencing was performed as described previously^7^. Subsequent filtering for rare variants with minor allele frequency <0.01 was performed in the gnomAD general population and subpopulation and in our internal cohort of approximately 2500 exomes using a custom filtering program. The results were checked manually using IGV Browser (https://software.broadinstitute.org/software/igv/). Filtering revealed a hemizygous missense variant in the *STAG2* gene (chrX:123179026, c.475T>C, p.Tyr159His) (Fig. 2A), while other rare variants could be excluded by phenotype and mode of inheritance. The research study was approved by the ethics committees at The University of Vienna, Austria and Varazdin General Hospital, Croatia. Biological materials from the patient and healthy donors were obtained with written informed consent in accordance with the Declaration of Helsinki. Pathogenicity predictions of the *STAG2* variant indicated a CADD-Phred score of 26.8, a PolyPhen of “probably damaging,” and a SIFT of “deleterious.” Furthermore, this variant has not been reported in the gnomAD or ClinVar database. Segregation within the family showed that both the healthy mother and sister are heterozygous for the variant and are therefore carriers of the disease (Fig. 2A). There was no family history of neurological disorders reported.

**Fig. 2:**
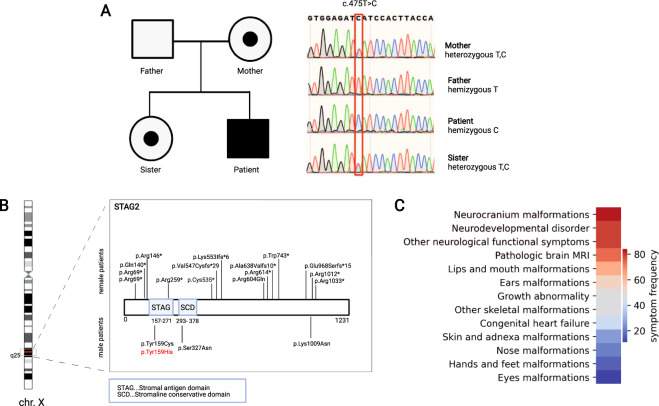
Mode of inheritance of this variant within the affected family and summary of all other reported variants. **A** Left panel—family pedigree, with squares representing males and circles representing females; dots represent carriers of the variant, and the affected patient is shaded in black. Right panel—Sanger sequencing chromatogram of all the analyzed family members, as labeled. **B** Summary of the reported pathogenic variants and their positions within the STAG2 protein. Our patient’s mutation is marked in red. **C** Heatmap of the common groups of symptoms described in the literature in patients with STAG2 mutations. Groups of symptoms arranged by frequency from most common to least common.

Since the first case of MKMS was described in 2015^9^, 17 other cases have been reported worldwide, of which only 3 are male (Fig. 2B). As *STAG2* is inherited in an X-linked manner with a wide variety of signs and symptoms (Fig. 2C), the severity of the phenotype seems to differ between male and female patients. It has been suggested that females, because of the second copy of the *STAG2* allele, can endure more severe mutations, while the same variants in males would lead to early embryonic lethality^1^. Consequently, females can show stronger phenotypes than males or, conversely, may not be affected by a mutation that leads to a phenotype in males. This is likely the case for the female family members of our index patient, who are healthy but are heterozygous carriers of the disease. Several healthy female carriers have also been described by Soardi et al.^3^, who reported an X-linked recessive inheritance pattern for a familial *STAG2* missense variant. The missense variants in the other two male patients reported are de novo mutations^4,5^.

In addition to differences in phenotype severity between males and females, there are differences in variant locations. The variants identified in male patients are localized mainly in functional domains of the protein (the STAG and SCD domains), with only one male patient having a variant more toward the C-terminal end of the protein and not in a known functional domain. The variants of female patients, on the other hand, are located in a cluster-like fashion at different positions in the gene and are not limited to known functional domains (Fig. 2B). We therefore hypothesize that, for male patients, missense mutations in the functional domains or in amino acids essential for functionality are sufficient to cause a phenotype, while in females, the second copy of the gene can compensate for this effect.

Due to the truncating variants in females, the severity of their MKMS symptoms can be greater than that of males, but there also seem to be differences in the types of symptoms reported in females and males. In general, females display a broader variety of symptoms, while male patients either show fewer symptoms or have not been described in the same level of detail (Table 1). Symptoms described only in female patients thus far are brachycephaly, long curly eyelashes, anteverted nares, long, smooth philtrum, downturned mouth, hypoplastic nails, hirsutism, cutis marmorata, strabismus, congenital diaphragmatic hernia, pulmonary hypoplasia, gastroesophageal reflux, rib fusion, vertebral abnormalities, single transverse palmar crease, and abnormal echocardiograms (Table 1). As new male case reports become available, the sex differences in the manifestation of MKMS will be clarified.

The clinical features of the patient reported by us closely overlap with other cases, with key symptoms including intellectual disability, developmental delay, short stature, hypotonia, micrognathia, dysmorphic facial features, and dysmorphic ears (Table 1). However, polymicrogyria, bilateral pes planus, and broad fingers and toes with soft dorsal surfaces and deeply inserted nails have not yet been reported in MKMS. It is interesting to note that our patient shared distinct features with a male patient reported previously (patient 11)^4^, who had a missense mutation at exactly the same position in the functional domain STAG (p.Tyr159Cys). Both patients initially showed a minimal patent foramen ovale (PFO) in an echocardiogram, which was found to have closed during a follow-up visit. Additionally, brain MRI in both patients revealed an ectopic posterior pituitary with a short, thin pituitary stalk. These common findings suggest that the position of the variant may contribute to the details of this phenotype in these two cases. Distinct symptoms such as minimal PFO and ectopic posterior pituitary with abnormal pituitary stalk could therefore reflect a dysfunction of the STAG domain due to the deleterious variants localized there. Interestingly, there were two reported female patients with the same c.205C>T/p.Arg69* variant that did not show any special common features. In female patients, differences in X inactivation might increase phenotypic variability, and these two p.Arg69* cases showcase the high variability of the disorder. It is tempting to postulate that clinical features in males may be less diverse than those in females because they are not influenced by X inactivation.

More common congenital abnormalities were described for hands and feet (Table 1). The severity of the bilateral pes planus is specific to our patient, which, possibly in combination with his polymicrogyria, renders him unable to stand or walk unaided. Additionally, the patient has broad fingers and toes with soft dorsal surfaces and deeply inserted nails, which have not been described in any other case. Notably, our patient presents with a unique neuroanatomical symptom in the form of polymicrogyria in the temporal and parietal lobe of the left hemisphere. Epilepsy was reported in only two other patients^4^ and may, in our patient, originate from polymicrogyria since this defect is often linked to seizures. Including our patient, seizures have been noted in only 3/19 cases of MKMS but are more frequent among patients with increased STAG2 expression (Xq25 duplication syndrome, OMIM#300979)^10^.

MKMS is a rare and highly variable disease, additional cases of which will shed light on sex-dependent clinical differences and possible variant-specific symptoms.

## HGV database

The relevant data from this Data Report are hosted at the Human Genome Variation Database at 10.6084/m9.figshare.hgv.3095.
